# Links between Breast and Thyroid Cancer: Hormones, Genetic Susceptibility and Medical Interventions

**DOI:** 10.3390/cancers14205117

**Published:** 2022-10-19

**Authors:** Man Lu, Hanqing Liu, Bilian Zheng, Shengrong Sun, Chuang Chen

**Affiliations:** Department of Breast and Thyroid Surgery, Renmin Hospital of Wuhan University, Wuhan 430060, China

**Keywords:** breast cancer, thyroid cancer, second primary cancer, etiology, risk factors, treatment

## Abstract

**Simple Summary:**

Breast and thyroid cancer are commonly diagnosed in women. Physicians have recognized and evaluated the phenomenon of two cancers occurring synchronously or asynchronously. The potential mechanisms are complex and various. Hormone, autoimmune attack and genetic predisposition are significant and shared factors influencing two cancers. Medical interventions for the first cancer and other life-related factors are reviewed and discussed, as well. This article aims to expound the relationship between breast and thyroid cancer, and suggests that physicians should monitor for the second cancer appropriately whenever one cancer occurs.

**Abstract:**

Breast and thyroid glands are two common sites of female malignancies. Since the late 19th century, physicians have found that the cancers in either thyroid or mammary gland might increase the risk of second primary cancers in the other site. From then on, many observational clinical studies have confirmed the hypothesis and more than one theory has been developed to explain the phenomenon. Since the two glands both have secretory functions and are regulated by the hypothalamic–pituitary axis, they may share some common oncogenic molecular pathways. However, other risks factors, including medical interventions and hormones, are also observed to play a role. This article aims to provide a comprehensive review of the associations between the two cancers. The putative mechanisms, such as hormone alteration, autoimmune attack, genetic predisposition and other life-related factors are reviewed and discussed. Medical interventions, such as chemotherapy and radiotherapy, can also increase the risk of second primary cancers. This review will provide novel insights into the research designs, clinical managements and treatments of thyroid and breast cancer patients.

## 1. Introduction

Breast cancer (BC) and thyroid cancer (TC) are among the most commonly diagnosed cancers in women, ranking 1st place and 7th place, respectively [1]. Since the late 19th century, the association of the two cancers has been recognized and evaluated by many researchers [2,3]. The earliest study in this field using epidemiological methods was published in 1984 [4]. Ron et al. collected data from 1618 women with primary TC and 39,194 women with primary BC. The standardized incidence ratios (SIR) were 1.68 and 1.89 for TC following BC and BC following TC, respectively. Although the elevated risks have been challenged by some other retrospective cohort studies [5,6,7,8], the discrepancy might be explained by different population screening programs, more accurate screening techniques and selection bias. More recently, data from multi-national large-cohort studies have demonstrated increasing risks with significant differences for TC in patients with primary BC, and vice versa [9,10,11]. A meta-analysis [12] further demonstrated that the odd ratio of secondary TC after BC was 1.55 (95%CI: 1.44–1.67). The odd ratio was somewhat lower for secondary BC after TC (1.18, 95%CI: 1.09–1.26). These studies and their associated standardized incidence ratios are summarized in Supplement Tables S1 and S2. And the level of evidence of these studies cited above is listed in Table S3. Interestingly, the incidence of TC has been revealed to increase in patients with other sex-hormone-related diseases, such as uterine fibroids and benign breast disease [13,14]. Additionally, the elevated risk of BC was also observed in those with autoimmune thyroid diseases [3,15]. Taken together, we can conclude with evidence that TC and BC can mutually increase the risk of each other.

The major question is “what is the mechanism”. Multiple explanations have been proposed by researchers. First, the two glands have secretory functions and are both regulated by the hypothalamic–pituitary axis, implying that they could be influenced by the same hormones (e.g., thyroxine and estrogen) [3,16,17,18]. Autoimmune attack to the thyroid gland, which increases the risk of TC, may also play a role in the oncogenesis of BC. Second, TC and BC share some alteration of geneses of in common, such as PTEN [19], KLLN [20], SDHx [21], PARP4 [22], MANCR [23,24] and VEGF [25]. Thus, genetic susceptibility is believed to cause co-occurrence of BC and TC [26,27,28]. Third, some medical interventions can increase the risk for secondary malignancies. Patients with advanced TC usually receive radioiodine therapy, according to the guideline. External beam radiation and chemotherapy are common therapies for progressed BC. These treatments are assumed to produce intracellular reactive oxygen species, to damage cells and to cause secondary cancers [29,30,31]. Finally, some other factors, including surveillance bias, obesity and diabetes mellitus, may also play roles in the co-occurrence of TC and BC.

**Figure 1:**
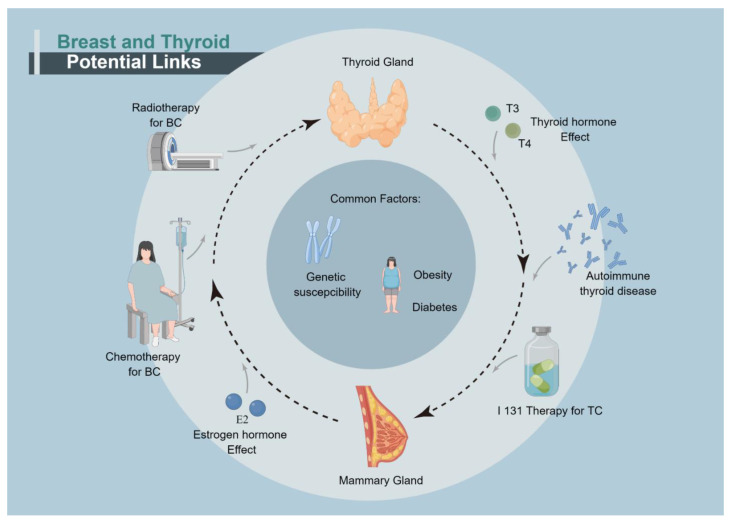
Summary of the potential links of breast and thyroid cancer. Abbreviation: TC: Thyroid Cancer; T3: Triiodothyronine; T4: Thyroxine; BC: Breast Cancer; I 131Therapy: Radioactive iodine therapy.

The aim of this article is to give a comprehensive review of the association between TC and BC and putative mechanisms (Figure 1).

## 2. Hormones and Their Receptors

### 2.1. Thyroid Hormones, Thyroid Hormone Receptor and BC

Thyroid hormones (TH) exert critical effects on skeletal growth, basal metabolism, nervous system development and cell proliferation and differentiation. The mammary glands are target tissues for THs, and their effects are complex. Postsurgical hypothyroidism is a common complication in thyroid cancer patients. Previous studies have suggested that TH dysfunctions, such as hyperthyroidism and hypothyroidism, can affect the risk of glandular epithelium-derived carcinomas [32].

In 1976, Kapdi and Wolfe found for the first time that there was a higher risk of breast cancer in those who received thyroid supplements due to hypothyroidism [33]. From then on, many studies have been performed over the last several decades, and the association between TH and BC remains inconclusive. Data from preoperative observational studies showed that TH and thyrotropin (TSH) are significantly associated with the risks of overall cancer, especially breast and ovarian cancer [15,34,35,36]. Søgaard and colleagues studied a large population-based cohort in Denmark, which recruited 61,873 women diagnosed with hypothyroidism and 80,343 women diagnosed with hyperthyroidism [3]. Standardized incidence ratios (SIRs) of BC increased in women with hyperthyroidism (SIR: 1.11, 95%CI: 1.07–1.16) and slightly decreased in women with hypothyroidism (SIR: 0.94, 95%CI: 0.88–1.00). Moreover, during the follow-up of more than 5 years, the SIRs in women with hyperthyroidism were further elevated (SIR: 1.13, 95%CI: 1.08–1.19), while the SIRs showed no significant difference in women with hypothyroidism (SIR: 0.96, 95%CI: 0.88–1.04). Notably, there are some limitations in this study, such as short administration time and confounding lifestyle factors. Kim et al. examined serum TH concentration in 62,546 healthy Korean females over 40 years of age and 834 incident BCs were observed [36]. Compared to the normal free thyronine (FT4) group, the high FT4 group showed an increasing hazard ratio (HR) for BC (1.98, 95%CI: 1.02–3.83). The association was revealed in postmenopausal women as well. Patients were divided into three groups based on TSH values. Individuals with higher TSH were revealed to have lower risk of BC than those with lower TSH (HR: 0.68, 95%CI: 0.55–0.84). Another prospective study in Sweden showed that FT4 was correlated positively with BC, yet free triiodothyronine (T3) showed no association. The results were consistent with Kim’s study [15,37].

Nevertheless, contrasting results were also presented. By following 2775 women, the study by Kuijpens et al. [16] found that hypothyroidism (OR = 3.8, 95%CI: 1.3–10.9) and the use of thyroid medication (OR = 3.2, 95%CI: 1.0–10.7) were associated with the incidence of BC. Interestingly, among patients without thyroxine supplements, those with FT4 levels in the lowest tenth percentile (OR = 2.3, 95%CI: 1.2–4.6) and TSH in the lowest tenth percentile (OR = 2.9, 95%CI: 1.5–5.7) were both at a high risk of BC. The reason remained unclear. Another case-control study supported the hypothesis that hyperthyroidism and high TH within normal ranges could increase the risk of BC, while hypothyroidism is a protective factor [38]. Some other case-control studies demonstrated there was no significant relationship between BC and TH [39,40]. Recently, a meta-analysis [32] demonstrated that hyperthyroidism was associated with higher risk of BC (pooled risk ratio: 1.20, 95%CI: 1.04–1.38). But hypothyroidism did not increase the risk of breast cancer. Another meta-analysis [41] published in 2021 was in accordance with Søgaard’s study [41]. The study revealed that breast cancer occurred more commonly in hyperthyroidism (OR = 1.12, 95%CI: 1.08–1.16) and less frequently in hypothyroidism (OR = 0.95, 95%CI: 0.91–1.00). The level of evidence of these studies cited above is listed in Table S4. The available evidences have some limitations: the design of retrospective observational studies, residual covariate and detection bias. For instance, once diagnosed with thyroid dysfunction, patients are more prone to have medical visits, therefore, incident cancers are more likely to be detected.

**Figure 2:**
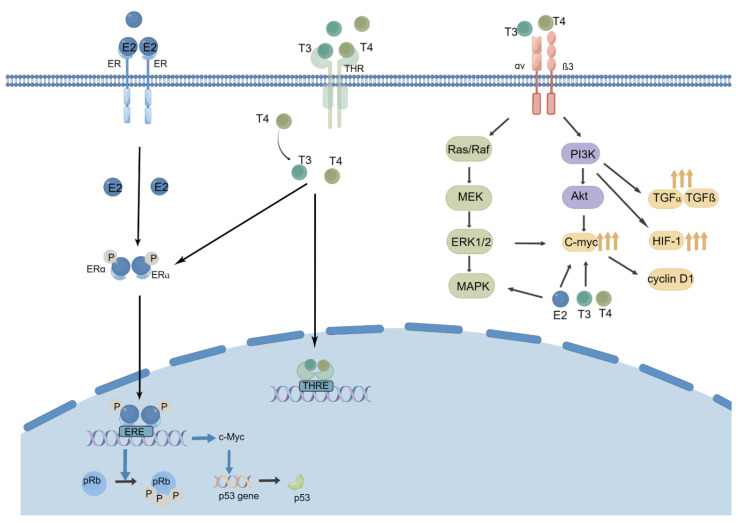
Thyroid hormone and estrogen-mediated signaling pathway.Estrogen and TH enhance nuclear localization of both THRɑ and ERɑ in breast cancer cells. Thyroid hormones facilitate the genomic effect of estrogen through ERa which bind estrogen response elements (ERE) [48]. Thyroid hormone and E2 regulate p53 and pRb(retinoblastoma protein) together (**Left**) [49]. TH can induce aberrant activation of MAP kinase and the PI3 kinase signaling pathways by binding ɑvß3 integrin as well. Metastasis and proliferation of BC is improved by increased C-myc, which is activated by the MAP kinase pathway [18]. TH could induce the high expression of hypoxia inducing factor 1 (HIF-1) and transform growth factor alpha (TGFα) in BC cell lines by activating the PI3K pathway (**Right**) [47].

The potential mechanisms between thyroid dysfunction and BC were studied by a relatively small number of studies [18,26,42,43,44,45]. The most proposed mechanism is that TH can initiate nongenomic actions via activating nuclear thyroid hormone receptor (THR) [46]. It can also cross-talk with other receptors, such as estrogen receptors (ER), progesterone receptors (PR), and human epidermal growth factor receptor 2 (HER-2) [42]. Firstly, Moretto FC demonstrated that T3 could induce the high expression of hypoxia inducing factor 1 (HIF-1) and transform growth factor alpha (TGFα) in the MCF7 breast cancer cell line by activating the PI3K pathway [47]. The process increases the aggressiveness and malignancy of BC. Secondly, TH can promote cell growth, which is similar to estrogen (E2). In Hall’s study [48], both T3 and E2 could promote cell proliferation in a dose-dependent manner in the MCF-7 and T47-D cell lines. Although the effect of TH was less strong, the activation of ERE-mediated gene expression in MCF-7 cells by T3 was proven. In BC cells with positive expression of ER, T3 treatment also increased the P53 level and induced Rb hyperphosphorylation, while an ER antagonist blocked these effects [49]. Additionally, T4 could also induce the serine phosphorylation of ERα, which then resulted in DNA binding and transcriptional activation. In addition, a significant crosstalk between the two hormones was proved. T3 enhances aerobic glycolysis (Warburg effect) of E2 in triple negative breast cancer cells, which was on behalf of transformed cells [50]. Finally, the fast signal pathway of avβ3, which mediates the balance between apoptosis inhibitors and promotors, was influenced by T3 [51]. Programmed death ligand 1 (PD-L1) gene expression was impacted by T4 via activating ERK1/2 [52] (Figure 2).

There are two subtypes of THR in human bodies: THRα and THRβ. They show adverse effects in the prognosis of BC patients. Heublein et al. found that THR was associated with BC patients with BRCA1 gene mutations [53]. THRβ showed a significant positive correlation with their five-year and overall survival rate, while THRα showed an adverse effect. In addition, according to a retrospective statistical analysis, higher expression of THRα1 showed a significantly worse disease free survival (DFS), while THRα2 expression may predict a better outcome of BC [43,45] Recently, Wahdan-Alaswad et al. [18] reported that TH treatment significantly and independently reduced disease-free and breast cancer-specific overall survival in steroid receptor positive BC patients in a long-term observational study. In their in vivo and in vitro experiments, TH treatment altered the nuclear colocalization of ER and THR. To conclude, THR and postsurgical thyroid dysfunction are important factors in the co-occurrence of BC and TC.

### 2.2. Estrogen, Progestin, and Its Receptors: ER/PR/HER-2 and TC

The most common cancer in women is BC, which is closely related to estrogen and progesterone. The prevalence of benign and malignant thyroid tumors occurs four or five times more in females than in males [54,55,56]. Notably, the difference is less obvious before puberty and after menopause [57]. Although the intrinsic causes of the gender discrepancy in thyroid disease have not been sufficiently elucidated, sex hormones are suspected to play a role.

Estrogen, a steroid hormone, exerts a pivotal impact on the regulation of body growth and the development of the immune system and reproductive organs. Patients with uterine fibroids, which are closely related to estrogen, had a significantly increased risk of thyroid cancer (HR = 1.64, 95%CI: 1.26–2.13), irrespective of whether or not they took myomectomy [14]. Persistence and recurrence of TC was significantly higher in patients who were diagnosed during pregnancy or within the second year after delivery, in comparison with the control group (*p* = 0.023) [58]. However, in a recent nationwide cohort study performed by Kim et al. in Korea [59], the finding did not support the theory that lack of estrogen is a protective factor. The risk of TC was higher after patients experience hysterectomy and oophorectomy, and there was no significant correlation between oral contraceptives and TC [60]. The level of evidence of these studies cited above is listed in Table S5.

**Figure 3:**
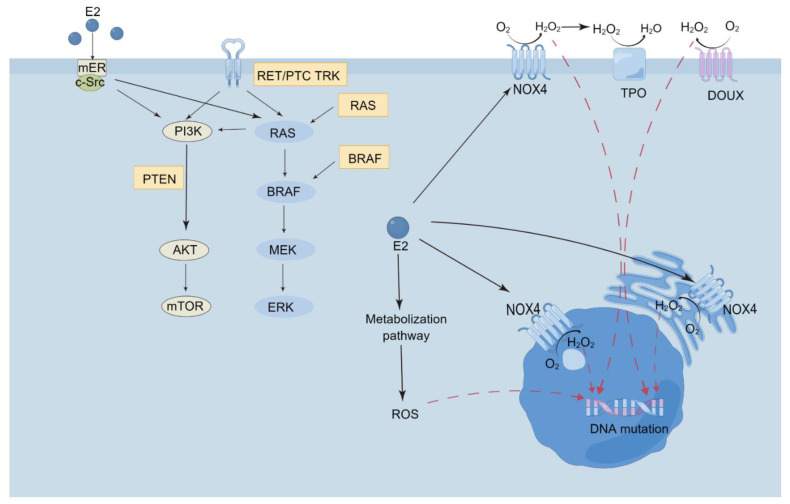
Estrogen non-genomic signaling pathway and estrogen-induced ROS generation in thyrocyte. The non-genomic signaling of E2 occurs via the membrane-bound receptor mER, which stimulates activation of the MAP kinase and the PI3 kinase signaling pathways (**left**). Due to the chromosomal rearrangement of the tyrosine kinase receptor TRKA, PET/PTC genes, BRAF gene, and RAS gene mutation, aberrant activation of the tyrosine kinase pathway occurs. Additionally, the PI3K AKT pathway may also be abnormally activated by mutational inactivation of PTEN. E2 stimulates these pathways [61]. In addition, E2 stimulates NOX4 to product ROS, as well as generates ROS through its own metabolization [65]. NOX4 located in the plasma membrane, endoplasmic reticulum, and nuclear membrane. ROS is able to reach nuclear, then promote some alterations which help thyroid carcinogenesis. DUOX: dual oxidase; E2: estrogen; NOX4: NAPDH oxidase 4; ROS: reactive oxygen species; TPO: thyroperoxidase.

These observative results were contradictory. Ex vivo studies provide more information. First, in the classical genomic pathway, the action of estrogen is mediated via two types of estrogen receptors, ERα and ERβ, which enhance DNA synthesis and proliferation. In another fast non-genomic pathway, a membrane-associated estrogen receptor (mER) is involved [61]. In the genomic pathway, estrogen enters the cell and transforms into estrogen-ER complex, which promotes the expression of target genes by binding an estrogen-responsive element (ERE) [62]. In the non-genomic pathway, E2 exerts function by mER, which activate MAPK and the PI3K signaling pathway. E2 also synergistically promotes the activation of the tyrosine kinase pathway in cells with RET/PTC fusion and BRAF mutation [61]. Second, thyroid cancer cells are replicated from mutated thyroid stem cells or progenitor cells, rather than primary thyroid cells [63]. In Xu’s study, the level of Erα in thyroid stem and progenitor cells was eight times higher than normal thyroid cells [64]. Third, Estrogen stimulates ROS production by NOX4, and ROS can reach the nucleus and contribute to thyroid carcinogenesis [65]. Mutual effect between thyroid redox homeostasis and estrogen in the development of thyroid carcinogenesis is another potential pathway [65] (Figure 3).

The expression of ER in thyroid epithelium cells is debatable. Some researchers failed to find ERs with immunohistochemical staining in normal thyroid tissue and cancer tissue [66,67]. Nevertheless, others detected ER on benign thyroid tissue [68]. With advanced techniques, ERα and PR expression was found by Vannucchi in 66.5% and 75.8% of patients, respectively, in 182 patients with papillary thyroid cancer [69]. It was the first time to find the occurrence of the ‘receptor conversion’ phenomenon in TC [69]. Overexpression of ERα in cancer tissue and lack expression of ERβ in surrounding tissue were reported in 2011 [70]. Low or lack of ER can be viewed as a hallmark of thyroid carcinomas. Heikkila found that ERβ expression was significantly lower in follicular thyroid cancer than in follicular adenomas [71]. Similarly, undifferentiated thyroid stem and progenitor cells expressed ERβ in a low level compared with differentiated human thyrocytes [64]. Therefore, ER expression in a low level may indicate dedifferentiation in thyroid cancer [72,73]. Tafani et al. found that HIF-1 and kB (NFkB), which mediate the immune and inflammation process, helped to transform thyroid cells to the malignant phenotype [74]. Importantly, HIF1a also regulated the ERα expression [75]. Tafani et al. proposed a hypothesis that ERα linked the two transcription factors in the progression of thyroid cancer [74].

HER-2 is an essential marker in the molecular target and prognosis of breast cancer. HER-2 expression in TC was also debatable, varying between 0% and 83.8% [76,77,78,79,80]. In 2001, Kim et al. found that HER-2 overexpression in thyroid cancer (83.8%) was more frequent than in benign tumors (16.7%), and was related to the reduction of PTEN protein [76]. Ruggeri et al. analyzed the HER-2 expression in differentiated thyroid cancer (DTC). The HER-2 overexpression was found in 44% (20/45) of the patients with follicular thyroid cancer (FTC) and 18% (8/45) with papillary thyroid cancer (PTC). After a median nine-year follow-up, six patients developed metastatic disease and five of them had HER-2-positive tumors [77]. The results from Tang et al. was consistent with Ruggeri’s study [78]. The positive HER-2 expression (45.71%) in PTC was significantly associated with lymph node metastatic (*p* < 0.05). Nevertheless, contradictory results are reported, as well. In Apostol’s study [79], the HER-2 expression was proposed to be a low risk factor. HER-2/neu positivity was found in twenty-five (20.8%) cases with twenty cases of high-risk and five cases of low-risk. Additionally, Mdah et al. demonstrated that the positive rate of HER-2 was just 6.9% in PTC [80]. The possible reasons for the HER-2 expression discrepancy might be varied size of sample, the general characteristic differences and the scoring methods. Large and independent researches with a unified estimated method of HER-2 are needed to provide more information.

## 3. Autoimmune Thyroid Disease and BC

Autoimmune thyroid diseases (ATD) include Graves’ disease and Hashimoto’s thyroiditis. The two specific biomarkers for Hashimoto thyroiditis, thyroid peroxidase antibody (TPOAb) and thyroglobulin antibody (TgAb), present in 90% patients [81]. Thyroid-stimulating hormone antibody (TSHRAb), which can bind with TSHR and increase the synthesis and release of TH, is a specific biomarker for Graves’ disease. Broadly speaking, the proposed mechanisms between ATD and cancer can be explained in two ways. On one hand, disordered immune system fails to eliminate cancerogenic cells. On the other hand, damaged immune system is prone to attack normal cells and abnormal cells indistinctively [82].The high rate of BC in ATD women was observed in 1975 [83]. Researchers recognized 18 BCs in 1810 cases with Hashimoto’s thyroiditis which was far more than the expected number (3.19 cases). The relationship between ATD and BC has been researched since then.

TPO is a member in the family of mammalian peroxidases. The family includes lactoperoxidase, myeloperoxidase, and so on. TPO express weakly in breast tumor and peritumoral tissue [84,85,86]. In 1996, Giani [87] evaluated 102 BC patients and 100 age-matched control healthy women. All patients experienced iodine deficiency. Hashimoto’s thyroiditis was found in 13.7% of BC patients and in only 2% of the controls (*p* < 0.005). And the detection rate of TPOAb was higher in BC patients than in the controls (23.5% vs. 8%, *p* < 0.005). They postulated that iodine deficiency might be an important factor in the oncogenic process. Subsequently, other studies recruiting BC patients showed similar results, demonstrating that TPOAb were detected more frequently in BC patients than in the controls [88,89,90,91,92]. Giustarini [90] detected TPOAb in 36 BC patients, 25 with breast benign disease (BBD) and 100 healthy women. The prevalence of TPOAb in BC patients (12/36, 33.33%) was significantly higher than in BBD patients (5/25, 20%) (*p* < 0.01) and in the controls (8/100, 8%) (*p* < 0.01). Since the study was retrospective, the diseases sequence remained unclear. A prospective study was conducted by Kuijpens in 2005 [16]. An unselected cohort of 2775 women around menopause was screened for TPOAb in 1994. There was an independent relationship between BC and the detection of TPOAb (OR = 3.3, 95%CI: 1.3–8.5). Contrary to expectation, the presence of TPOAb showed no relationship with BC after 9 years follow-up (OR = 1.1, 95%: 0.4–2.7). These results were in line with Hedley’s study [93], which revealed no correlation between ATD and BC. TPOAb may be the first sign of thyroiditis induced by irradiation or chemotherapy for BC. Another explanation is that the presence of TPOAb is the immune response of the tumor itself. Third, patients’ fear and stress due to BC may influence the immune system [16]. Recently, a meta-analysis showed that TPOAb presence was significantly associated with an increased risk of BC [94] (OR = 2.86, 95%CI: 2.17–3.77, *p* < 0.001). In summary, a correlation exists between TPOAb and BC. The discrepancy between studies might be explained by the small sample size, the different racial make-up of the study cohort, the diagnosis criteria, and lifestyle differences. A large prospective cohort study is needed to ascertain the association between TPOAb and BC.

TgAb is another indicator for ATD. Similar results have been presented. Some cross-sectional studies confirmed the link between TgAb and BC [90,91]. Giustarini [90] showed that the rates of TgAb in the BC patients (33.33%) was higher than of the BBD patients (16%, *p* < 0.01) and in the controls (12%, *p* < 0.01). However, contrary results were also demonstrated. The detection of TgAb was higher in BC patients, without significant difference (16.6% vs. 12%) [87]. The possible reason might be the small sample size. A prospective study is lacking. A meta-analysis in 2020 [94] demonstrated that TgAb was associated with BC (OR = 2.71, 95%CI: 1.81–4.05, *p* < 0.001). Thus, ATD shows a connection with BC. Further and prospective studies are also required.

Few studies have evaluated the relationship between TSHRAb and BC. In 2013, Szychta [95] found that Graves’ disease and BC was statistically correlated. This study detected TSHRAb in 9 BC patients, 47 BBD patients and 1630 women hospitalized for several non-oncological diseases. The mean values of serum TSHRAb were statistically higher in BC patients than in the controls (*p* = 0.0006). In univariate regression analysis, breast cancer had a predictive value for TSHRAb (OR = 1.10, 95%CI: 1.01–1.20, *p* = 0.0222). In addition, TSHR expression is common in BC (34/44 cases, 77%) [96]. No unanimous conclusion has been drawn concerning TSHRAb in BC patients. The shortcoming of Szychta’s study is that the sample was too small to trust. Large and prospective research is required to verify the association of TSHRAb and BC. The level of evidence of these studies cited above is listed in Table S6.

Moreover, sclerosing lymphocytic lobulitis (SLL), a rare benign disease of the breast, was viewed as an autoimmune disease of the breast and strongly associated with other autoimmune disorders [97]. SLL of breast and thyroid microsomal antibodies was first reported by Soler and Khardori in 1984 [98]. Dubenko, M. reported a case described the association of SLL of the breast with Graves’ disease [97]. Park also published a case-report about SLL of breast in patient with Hashimoto’s thyroiditis [99]. Interestingly, compared to healthy people, lobulitis was encountered in 21 of 41 (51%) patients at hereditary high risk of BC [100]. Recently, Hieken found moderate or severe lobulitis was more common in BC (73%) than benign disease (13%) (*p* = 0.003) [101]. Published case-reports about SSL of the breast and ATD are few. But the viewpoint that SLL of the breast was strongly linked autoimmune disorders was common in many authors. More studies concentrating on SLL and ATD are needed to illuminate the issue.

## 4. Iodine, Sodium Iodide Symporter and BC

Thyroid and breast both need iodide to produce iodoproteins, which participate in the biosynthesis of TH and breast milk as a source of neonatal nutrition [102].

There is also an interesting observation that the breast cancer incidence was relatively low in Japanese women. This is likely due to their seafood-rich diet [103]. Funahashi’s study revealed that Lugol’s iodine or iodine-rich Wakame seaweed is a protective factor in rats with BC [104].

Sodium iodide symporter (NIS), a membrane-bound glycoprotein, is located in the basolateral cell membrane. Its function is to transport and accumulate iodide ion (I−) into cells. NIS mediates the active uptake of I− in the thyroid, which is the crucial step in thyroid hormone biosynthesis. Other than the thyroid, NIS can mediate I− uptake in other tissues, such as salivary glands, gastric mucosa, and lactating glands. More than 80% BC samples and 23% peri-tumor breast samples are observed to express NIS [105]. Benign breast diseases, such as fibroadenoma, show a higher expression of NIS proteins and accumulation of iodide [106,107]. Thus, it is difficult to regard NIS as a specific indicator of BC [17,105]. Relatively higher expression of NIS does not indicate higher iodide uptake due to the dislocation in cytoplasm rather than cell membrane. Another explanation of this phenomenon is that the NIS may be overestimated due to the defective method of detecting NIS protein with polyclonal NISAbs [17,108].

## 5. Oncogenic Effects of the Therapies for Primary Cancer

### 5.1. Radioactive Iodine Therapy and Breast Cancer

Since the 1940s, radioactive iodine (RAI) has been used in the treatment of hyperthyroidism. Postsurgical RAI therapy is also used in patients with cervical lymph node metastasis, distant metastasis and extrathyroidal extension. RAI can be transported into thyroid epithelium cells via NIS to perform the tumoricidal effect. NIS is also found to be expressed in the breast, salivary lacrimal gland, ovaries and gastric mucosa [109]. Furthermore, BC cells have been proven to have functional expression of NIS [110]. Concerns have been raised with respect to radioactive iodine therapy, given the possible increased incidences and mortality of second primary malignancy, including breast cancer.

Some studies shed light on evaluating cancer risk after RAI, and their results are conflicting [111,112,113]. The large long-term follow-up analysis by Kitahara [114] included 18,805 patients. The study revealed that for every 1000 patients after RAI therapy, an estimated 18 to 30 deaths due to solid cancer would occur (4 to 6 were BC). A larger part of these deaths would occur more than 20 years after RAI treatment. By developing a biokinetic model [115], the high-quality, individualized organ- and tissue-specific dose estimation were obtained. The association between RAI treatment and the mortality of BC was revealed (RR = 1.12, 95%CI: 1.003–1.32, *p* = 0.04) at the dose of 100-mGy. However, the study did not take covariates into considerations, such as smoking, obesity and alcohol use and concomitant diseases. In 2017, Silva-Vieira [116] evaluated second primary cancer (SPC) incidence in 2031 patients with/without DTC receiving RAI treatment, with a median follow-up period of 8.8 years. A total of 130 SPC were diagnosed and the most common cancer was BC (31%). Compared to control groups, a statistically significant high risk of SPC in RAI treatment was found (RR = 1.84, 95%CI: 1.02–3.31, *p* = 0.026). Notably, an increasing incidence of SPC by year was revealed. The 10-year cumulative incidence rates of SPC in groups who received 0, <100, 100–199, 200–299, and >300 mCi were 4.4%, 3.9%, 7.5%, 11.8%, and 10.9%, respectively. Compared to no RAI group, the relative risk of SPC in 200–299 and >300 mCi group was 2.43 (95%CI: 1.17–5.01) and 2.29 (95%CI: 1.03–5.08), respectively. The incidence of breast cancer in patients with thyroid cancer receiving RAI treatment increased compared to the controls. Yet there was no significant elevation compared different cumulative dose of RAI [29]. The author postulated that increasing sodium-iodide symporter (NIS) expression before RAI is responsible for carcinogenesis, rather than radiation exposure. Chen proposed that immortal time bias might bring about false results [117].

Different opinions on postsurgical RAI in thyroid cancer patients still exist [113,118]. Recently, a meta-analysis showed no statistically significant elevations in the risk of SPC (OR = 1.02, 0.95–1.09) [119]. However, in a dose-response analysis based on two original studies, RAI was significantly associated with BC (*p* = 0.03) [119]. This study suggested that only high cumulative doses led to increased risk of SPC. Thus, the dose of RAI is needed to take into careful consideration in clinical practice. And more researches exploring rational and safe dose of RAI is needed. Because of the lengthy latent period of some solid cancers, a long-term follow-up is required. As the most common of SPC, BC may have particular mechanisms related to RAI.

### 5.2. Chemotherapy and Thyroid Cancer

Chemotherapy is a conventional and crucial treatment for breast cancer. The application and dose of chemotherapeutic drugs is under strict management, due to their potent adverse effect on normal organs and cells [120]. Therefore, thyroid function seems to be vulnerable to chemotherapeutic drugs. Normal thyroid function may be impacted due to cell death. Another theory suggested that hypothalamus–pituitary–thyroid axis down-regulation was an adaptation to adverse physical conditions in ill patients [121]. A low TH level of protects the body against tissue damage by down-regulating cellular metabolism [122].

A number of studies found that thyroid dysfunction occurs in patients who receive systemic chemotherapy. However, no unanimous conclusion was reached. Kailajärvi [123] performed a study testing FT3, FT4 and TSH in 15 women receiving cyclophosphamide, methotrexate and 5-fluorouracil chemotherapy. The results revealed that T3 and T4 declined temporarily but the TSH level had no alteration. Kumar [124] designed a prospective observational study to evaluate TH change in 198 BC patients. Patients received systemic chemotherapy agents of cytoxan, adriamycin and 5-fluorouracil. A significant reduction in mean serum T3 uptake (*p* < 0.05) was observed. Other studies show that T3 and T4 declined but TSH increased [122,125]. In 2015, de Groot [122] obtained serum samples at baseline, before the 2nd chemotherapy cycle, and at end of neoadjuvant treatment with docetaxel, doxorubicin and cyclophosphamide. FT4 levels decreased (*p* = 0.0001) and TSH levels increased significantly (*p* = 0.019). It is reasonable that lower FT4 level was regarded as a protective response [122]. A systematic review showed that T3 and T4 levels decreased after chemotherapy but TSH remained unchanged [126]. Hence, a routine screening for TH needs to be taken into consideration in BC patients after chemotherapy. Although there are studies focusing on thyroid dysfunction, few researches have evaluated the incidence of second primary cancer and different chemotherapy regimens. The potential reasons might be that chemotherapeutic agents aims to kill malignant cell and microenvironment of systematic treatment is not appropriate for cancer growth and invasion.

### 5.3. External Beam Radiation, Mammography and Thyroid Cancer

Postoperative radiotherapy for breast cancer can decrease the risks of local recurrence and death. This adjuvant treatment is used in patients receiving breast conserving surgery (BCS) and mastectomy with axillary lymph node metastasis. With a mean 5-year relative survival rate of over 80%, the majority of women were observed to have one or more treatment-induced SPCs. The balance between the expected benefit and the risk of SPC needed attention. Thyroid cancer and radiation have close relation. Data from Chernobyl showed that the risk of thyroid cancer in neighborhood has increased since the accident [127,128]. Besides, patients with Hodgkin’s lymphoma who received radiotherapy above the diaphragm have a a higher risk of second thyroid cancer [129,130].

Radiotherapy for breast cancer inevitably exposes the adjacent normal organs to unwanted radiation, with a gradual dose-fall outside the field-edge [131]. A national population-based study including 46,176 patients treated for early BC was conducted by Grantzau’s team in 2013 [132]. They classified SPC into two groups: radiotherapy-associated sites and non-radiotherapy-associated sites. The former involves esophagus, lung, heart/mediastinum, pleura, bones, and connective tissue. The other cancers, thyroid, stomach, liver and so on, are included in another group. The SPC of the first group showed an increased risk (HR = 1.34, 95%CI: 1.11–1.61) and the HR was even higher when the follow-up period was extended to 15 years. There was no increased risk for the second group (HR = 1.04, 95%CI: 0.94–1.1). The thyroid gland was exposed to considerable doses (>26 Gy) in breast radiation treatment in a retrospective study [133]. In 2017, Burt [134] used the SEER database to obtain data from 374,993 BC patients. A total of 154,697 patients received external beam radiation. The rate of SPC was significantly greater than the endemic rate in BC patients without radiation therapy (SIR = 1.2, 95%CI: 1.19–1.22). However, the rates of second primary thyroid cancer in radiation group showed no statistical significance (SIR = 1.09, 95%CI: 0.97–1.22). A systematic review and meta-analysis including 522,739 patients revealed that pooled incidence of second TC at ≥15 years after irradiation for BC were 3.15 [30]. The risk of second thyroid cancer for non-radiated patients increased without significant difference [30]. In summary, there was no evidence to support the theory that patients receiving radiation are at higher risk for TC.

The incidence of hypothyroidism increases in BC patients after postoperative radiotherapy [135,136,137]. A study aimed to assess the association of different radiation targets with hypothyroidism [135]. By comparing groups of regional node irradiation (supraclavicular lymph node) and breast only, the cox model revealed an adjusted hazard ratio of 2.25 (95%CI, 1.49–3.38). The risk was more prominent in patients aged under 60 years. Thus, a routine examination of the thyroid is needed, especially 10–15 years after the initial treatment of breast cancer radiation. Physicians need to balance the expected benefits and long-term side effects after radiation in patients with early-onset BC.

Concern over thyroid exposure during mammography has been expressed. Thyroid shields are supposed to protect during mammography. Sechopoulos [138] demonstrated that thyroid exposure had a maximum average effective dose of 0.13 μSv from digital, and 0.17 μSv from film-screen mammography. The cumulative lifetime risk of TC is approximately 56 per billion (or 1 in 17.8 million) if individuals received serial annual screening mammography examinations between the ages of 40 and 80 years. This effect to the thyroid is negligible. In a large population-based study [139], there was also no evidence supporting the higher risk of TC in the group who received mammography (HR = 1.2, 95%CI: 0.81–1.77). Therefore, mammography is unlikely to be a possible factor increasing the risk of TC. The level of evidence of studies cited about Oncogenic effects of the therapies for primary cancer is listed in Table S7.

## 6. Genetic Predisposition

Not all second primary cancers are ascribed to molecular immunization environment, hormones or oncogenic effects of the therapies for the first cancer. Some sporadic cases may be attributed to genetic susceptibility and lifestyle factors. A large retrospective study among twins in Nordic countries revealed that hereditability accounted for approximately 33% of cancer risk [140]. A familial link between thyroid and breast cancer was found by a study based on the Swedish Family-Cancer Database [141]. The incidence of thyroid cancer increased in individuals who have ≥ two first-degree relatives with BC (RR = 1.90, 95%CI: 1.38–2.63). The associations between ovarian and prostate cancers are known because of the BRCA1/2 mutation and hormonal effects. But the genetic association with BC and TC was novel with the current statistical support [141].

PTEN hamartoma tumor syndrome (PHTS, comprising Cowden, Bannayan-Riley-Ruvalcaba, and Proteus-like syndromes). PHTS are featured as an increased risk for several solid malignances, including breast cancer, thyroid cancer, colorectal cancer, endometrial cancer and melanoma [142]. The standardized incidence of BC and TC ranged from 6 to 9 [143]. PHTS is due to germline mutations of tumor suppressor gene: phosphatase and tensin homolog (PTEN). It inhibits the catalytic activity of the enzyme PI3K and then upregulates the PI3K-AKT pathway, which facilitates the survival, proliferation and migration of tumor cells [19,144]. In a 7-year multicenter prospective study [143], 2024 patients with invasive cancer histories were included and 5.6% of them were detected to have PTEN mutations. Compared to the general population, the risk of second BC and TC significantly increased (SIR = 8.92, 95%CI: 5.85–13.07; SIR = 5.83; 95%CI: 3.01–10.18). Additionally, PHTS can be induced by mutations of KLLN and the succinate dehydrogenase complex (SDHx). SDHx participates in the composition of mitochondrial complex II, which transports electrons in Kreb’s cycle. SDHx mutations can dysregulate TP53, which induce apoptosis by upregulating the proteasomal degradation of p53 [21]. In Ni’s research [21], germline variations of SDHx occurred in 8% (49/608) of individuals with no PTEN mutation and 6% (26/444) of individuals with Cowden syndrome. It was not found in the control group of 700 individuals. Of note, the group with only SDHx mutation showed the highest TC incidence. KLLN, a transcription factor, shares a bidirectional promoter with PTEN and encodes KLLN protein [20].Patients with a KLLN mutation had a 3-fold higher incidence of BC (*p* < 0.0001). Ngeow et al. showed that the SIR of TC was 45 for KLLN mutation in individuals with PHTS (95%CI: 26–73, *p* < 0.001) [145]. New variants and mutations will continue to be identified.

In addition, PARP4 is another shared factor in patients with both BC and TC [22]. PARP4 encodes poly-ADP-ribose polymerases (PARPs) and is an important component of DNA repair. In 2016, Ikeda [22] found that the variants in PARP4 gene were detected at high frequency (OR = 5.2, *p* = 0.00001) in genomic analysis among 14 patients with co-occurrence of BC and TC. Epigenetic changes in the genome are worth taking into account. Long non-coding RNAs (lncRNA) named MANCR (mitotically-associated long noncoding RNA), which play a role in cell proliferation, viability, and genomic stability, are important regulators in the genomic stability of aggressive breast cancer [24]. Many long noncoding RNA(lncRNAs) are differentially expressed in TC tissues when compared with normal tissues [23]. Angiogenesis in patients with both two primary cancers has been illustrated. Wei [25] found that the vascular endothelial growth factor (VEGF) was overexpressed in thyroid and breast carcinomas in 14 patients with BC compared with the benign breast disease group (*p* < 0.01). The incidence of microvascular angiogenesis in TC increased in patients with a history of BC. Thus, contrast-enhanced ultrasound is a promising tool to examine the thyroid gland if the patient has a history of BC. However, the sample of this study was small. Large and multicentric research is needed to verify this result.

**Figure 4:**
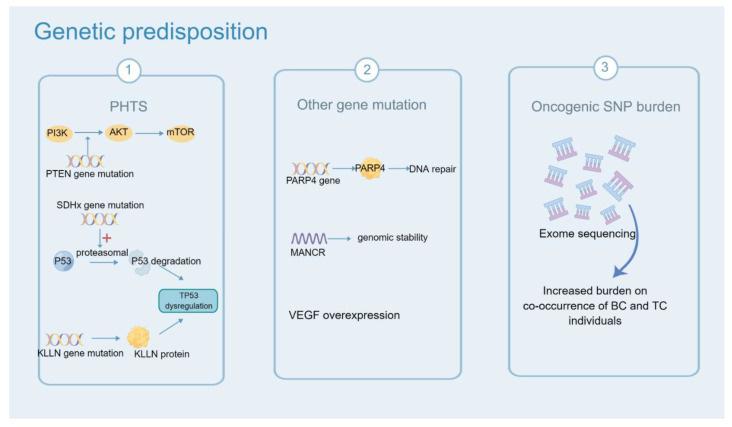
Summary of genetic susceptibility of BC and TC. In part 1, PTEN hamartoma tumor syndrome (PHTS, comprising Cowden, Bannayan-Riley-Ruvalcaba, and Proteus-like syndromes) is due to germline mutations of tumor suppressor gene: phosphatase and tensin homolog (PTEN). The gene mutation of PTEN upregulates the PI3K-AKT pathway. SDHx (the succinate dehydrogenase complex) mutations can dysregulate TP53 by upregulating p53 proteasomal. KLLN gene encodes the KLLN protein, which is a transcription factor. KLLN mutation influences TP53 dysregulation. In part 2, PARP4 gene encodes poly-ADP-ribose polymerases (PARPs) and is an important component of DNA repair. MANCR (mitotically-associated long noncoding RNA) is an important regulator of the genomic stability of aggressive breast cancer. The vascular endothelial growth factor (VEGF) was overexpressed in TC and BC. In part 3, increased oncogenic single nucleotide polymorphism (SNP) burden in co-occurrence of BC and TC.

Some cases were associated with monogenic disorders of autosomal inheritance, but the majority cases of co-occurrence of BC and TC was considered to be polygenic. A recent retrospective case-control study is in line with hypothesis of genetic predisposition [27]. Bakos [27] compared the genetic profile of 15 cases with breast cancer and 19 cases with co-occurrence of the two cancers by using whole exome sequencing. The level of evidence of studies cited about genetic predisposition is listed in Table S8. Increased oncogenic single nucleotide polymorphism burden was associated with co-occurrence of BC and TC. This research further confirmed that the genetic predisposition plays a significant role in tumorigenic progression (Figure 4).

## 7. Other Factors

The International Agency for Cancer Research has demonstrated the correlation between being overweight or obese and 13 types of cancer [146], including postmenopausal BC and TC. The obesity groups whose BMI ≥ 35kg/m^2^ were at the highest risk of invasive BC (HR = 1.58, 95%CI = 1.40–1.79) [147]. Obesity is also related to advanced BC, including larger tumor size, positive lymph nodes, regional or distant stage, and deaths [147]. Ewertz [148] found that being obese or overweight may represent a modifiable risk factor in BC occurrence and progressive. Engeland [149] reported that the rates of TC increased moderately with increasing BMI and height in males and females (RR = 1.29, 95%CI = 1.13–1.46). Kitahara [150] further demonstrated central adiposity influenced high incidence of TC. Some other types of studies supported the association between the risk of TC and obesity [151,152,153]. In 2013, Kim [154] reported a strong relationship between BMI and aggressive features, including larger tumor size (OR = 1.31, *p* < 0.001), advanced TNM stage (OR, 1.30, *p* = 0.003) and extra-thyroidal invasion (OR 1.23, *p* = 0.006). The great prognosis of TC after surgical therapy may be a possible reason that the harm of obesity is too weak to change it. Being obese or overweight are linked with the incidence and pathological features of TC.

It is well-known that excessive adiposity is able to progressively cause a series of co-morbidities. Park [155] found triple-negative BC was in a higher risk in the T2D group (HR = 1.40; 95%CI = 0.90–2.16). Interestingly, longer duration metformin use for T2D showed a protective role in ER-positive BC (HR = 0.62; 95%CI = 0.38–1.01; *p* = 0.09). Moreover, a study involving 8482 patients in 2021 [156], demonstrated that the prognosis of BC was better in patients who persisted in maintain a diabetes risk reduction diet. This diet decreased the risk of BC-specific mortality (HR = 0.80; 95%CI = 0.65–0.97; *p* = 0.02) and risk of all-cause mortality (HR = 0.66; 95%CI = 0.58–0.76; *p* < 0.0001). Meanwhile, the connection between diabetes mellitus and TC was observed. In a study that applied Mendelian randomization, the casual link between diabetes mellitus and TC was confirmed [157]. Compared to individuals in the lowest quartile for genetic liability of T2D, higher odds of TC were found in the highest quartile (OR, 1.45; CI, 1.11–1.90). Diabetes mellitus may be a conceivable factor connecting BC and TC. And the level of evidence of these studies cited above is listed in Table S9.

## 8. Conclusions

The association between BC and TC has been evaluated. Patients who have either cancer history are at an increased risk of the other second primary cancer compared to the general population. “What is the mechanism?”, this problem has been illustrated and explored partially. The shared common features may be the etiologies and possible causative factors of BC and TC. For example, the hormone effects of TH and E2, autoimmune attack, genetic predisposition and other life-related factors. However, some results remain inconsistent. Well-designed and large cohort studies are needed to prove the causative factors linking BC and TC. Further investigation into gene mutation and disordered gene expression underlying BC and TC development is promising. Complicated, different, and cross-talk signal pathways exploration is needed as well. On one hand, RAI therapy should be taken into consideration by clinicians when balancing the benefits and risks. On the other hand, systematic chemotherapy and partial external beam radiation can both affect the thyroid gland. Systematic chemotherapy and immunity therapy lack convincing evidence to support their relation with TC. Large cohort studies are needed to evaluate the oncogenic effect of external beam radiation on certain regions. Common tumorigenic pathways to BC and TC and shared risk factors can be screened. The studies on co-occurrence of BC and TC can reveal the biological behavior of two cancers and provide novel treatment strategies, which might guide clinical practice in the future.

## Supplementary Materials

The following supporting information can be downloaded at: https://www.mdpi.com/article/10.3390/cancers14205117/s1, Table S1: Standardized incidence ratios of thyroid cancer after breast cancer; Table S2: Standardized incidence ratios of breast cancer after thyroid cancer; Table S3: Level of evidence about Thyroid cancer and Breast cancer; Table S4: Level of evidence about Thyroid Hormone and Breast cancer; Table S5: Level of evidence about Estrogen and Thyroid cancer; Table S6: Level of evidence about Autoimmune thyroid disease and Breast cancer; Table S7: Level of evidence about Oncogenic effects of the therapies for primary cancer; Table S8: Level of evidence about Genetic predisposition; Table S9: Level of evidence about other factors and Breast cancer. And the level of evidence was classified by standard of OCEBM (Centre for Evidence-Based Medicine, University of Oxford, OCEBM). References [157,158,159,160,161,162,163,164,165,166,167,168,169,170,171,172,173,174,175,176,177,178,179,180,181,182,183,184,185,186,187,188,189,190,191,192,193] are cited in the supplementary materials.
