# Supplementary material for: Links between Breast and Thyroid Cancer: Hormones, Genetic Susceptibility and Medical Interventions
Source: Cancers (Basel). 2022 Oct 19;14(20):5117. doi: 10.3390/cancers14205117 (PMC9600751; doi:10.3390/cancers14205117)
Supplement: Supplementary file 1 [file cancers-14-05117-s001.zip › cancers-1942996-supplementary.pdf]

1. Level of evidence once discussing the impact of published manuscripts.

Each of published manuscripts was listed as followed. And the level of evidence was classified by standard of OCEBM (Centre for Evidence-Based Medicine, University of Oxford, OCEBM).

Table S1. Standardized incidence ratios of thyroid cancer after breast cancer.

Study	Location(s)	Study Period	Mean Follow-Up Period (y)	Case(n)		SIR (95% CI)
				BC	TC	
Jin 2022	Korea	2002–2013	NA	3308	84	1.91 (1.47–2.49)
Cieszyńska 2022	Poland	1996–2014	6.3	10,832	53	4.27
Bright 2019	England, Wales	1971–2006	14.3	36,236	35	1·7 (1·2–2·4)
Huang	China	1999–2013	5.4	13,978	247	6.92 (6.03–8.29)
Corso 2018	Italy	1994–2010	NA	21,527	78	1.54 (1.23–1.92)
Silverman 2017	Israel	1990–2006	10	46,090	141	1.46 (1.20-0-1.71)
Jung 2017	Korea	1989–2014	NA	3344	44	2.29 (1.67–3.08)
Hung 2016	Taiwan	1997–2011	4.28	101,493	183	2.15 (1.85–2.48)
Molina-Montes 2013	Spain	1985–2007	6.4	5897	8	1.01 (0.02–2.01)
Mellemkjær 2011	Denmark, Finland, Norway	1943–2006	8.8	304,703	347	1.41
Lee 2008	Taiwan	1979–2003	5.4	59,001	45	1.42 (1.04–1.90)
Kirova 2008	France	1981–1997	10.5	16,705	20	0.87 (0.53–1.35)
Raymond 2006	USA (SEER)	1973–2000	NA	335,191	314	2.23
Mellemkjær 2006	Europe, Canada, Australia, Singapore	1943–2000	7.2	525,527	471	1.62
Soerjomataram 2005	Netherlands	1972–2000	6.6	9919	2	0.8 (0.1–3.3)
Sadetzki 2003	Israel	1960–1998	7.3	49,207	59	1.34 (1.03–1.72)
Levi 2003	Switzerland	1974–2998	6.4	9729	6	1.01 (0.37–2.20)
Tanaka 2001	Japan	1970–1994	8.6	2786	7	3.7 (1.5–7.6)
Huang 2001	Canada	1973–1993	6.8	194,798	140	1.2 (1.0–14)
Evans 2001	England	1961–1995	5.7	145,677	37	1.05
Rubino 2000	France	1973–1992	9.5	4416	4	2.2 (0.7–5.2)
Volk 1997	Slovenia	1961–1985	7.3	8791	10	2.5 (1.2–4.6)
Doherty 1993	Scotland	1954–1964	18	3926	4	2.33 (0.63–5.95)
Murakami 1987	Japan	1965–1982	5.7	9503	9	3.2 (1.5–6.1)
Teppo 1985	Finland	1954–1979	NA	26,617	22	1.95
Harvey 1985	USA	1935–1982	6.6	41,109	28	1.6
Schenker 1984	Israel	1960–1977	NA	12,302	33	1.7

Abbreviations: BC, breast cancer; CI, confidence interval; n, number of cases (studies with n < 1000 excluded); NA, data not available; TC, thyroid cancer; y, years.

Table S2. Standardized incidence ratios of breast cancer after thyroid cancer.

Study	Location(s)	Study Period	Mean Follow-Up Period (y)	Case(n)		SIR (95% CI)
				BC	TC	
Jin 2022	Korea	2002–2013	NA	39	3949	1.67 (1.15–2.43)
Bright 2019	England, Wales	1971–2006	NA	170	6215	1.3 (1.1–1.5)
Lu 2013	Taiwan	1976–2006	7	102	19,068	1.42 (1.16–1.72)
Kim 2013	U.S.A	1973–2008	NA	1041	52,103	1.13 (1.06–1.20)
Consorti 2011	Italy	1996–2009	6.2	7	75	3.58 (1.14–8.37)
Mellemkjær 2006	Europe, Canada, Australia, Singapore	1943–2000	NA	552	NA	1.31 (1.21–1.43)
Verkooijen 2006	Netherlands	1985–1999	10.6	5	282	1.52 (0.46–3.18)
Berthe 2005	France	1960–1998	8	12	875	1.19 (0.62–2.08)
Rubino 2003	France, Italy, Sweden	1934–1995	13	128	6841	1.3 (1.0–1.5)
Adjadj 2003	France	1934–1955	12	48	2365	1.3 (1.0–1.7)
Sadetzki 2003	Israel	1960–1998	9.4	4911	70	1.07 (0.84–1.34)
Hemminki 2001	Sweden	1958–1996	NA	113	19,281	1 (0.8–1.2)
Edhemovic 1999	Slovenia	1971–1993	5.2	4	894	1.12 (0.31–2.87
Dottorini 1995	Italy	1960–1993	8	10	694	2
Glanzman 1992	Switzerland	1960–1988	14.5	5	298	1.9 (0.6–4.5)
Hall 1991	Sweden	1950–1975	16	24	1955	1.46
Johns 1989	U.S.A	1975–1985	2.7	5	115	8.9
Edmonds 1986	England	NR	11.2	6	258	2.37
Osterlind 1985	Denmark	1943–1980	5.9	11	1351	0.96 (0.76–1.20)
Ron 1984	USA	1935–1978	8	24	1618	1.89

Abbreviations: BC, breast cancer; CI, confidence interval; n, number of cases (studies with n < 1000 excluded); NA, data not available; TC, thyroid cancer; y, years.

Table S3. Level of evidence about Thyroid cancer and Breast cancer.

Study	Level of evidence	Year
Ron E et al. [1]	3	1984
Osterlind, A., et al. [2]	3	1985
Edhemović, I., et al. [3]	3	1998
Evans, H. S., et al. [4]	3	2001
Soerjomataram, I., et al. [5]	3	2005
Langballe, R., et al. [6]	3	2011
Mellemkjaer, L., et al. [7]	3	2006
Kim, C., et al. [8]	3	2013
Nielsen, S. M., et al. [9]	1	2016

Table S4. Level of evidence about Thyroid Hormone and Breast cancer.

Study	Level of evidence	Year
Tran, T et al. [10]	1	2020
Kapdi, C rt al. [11]	3	1976
Tosovic, A., et al. [12]	3	2012
Hellevik, A. I., et al. [13]	2	2009

Journy, N. M. Y., et al. [14]	2	2017
Kim, E. Y., et al. [15]	2	2019
Søgaard, M., et al. [16]	2	2016
Kuijpens, J. L. P., et al. [17]	2	2005
Ortega-Olvera, C., et al. [18]	4	2018
Bach, L., et al. [19]	4	2020
Freitas, Paula Andréa V. C. J et al. [20]	4	2016
Chen, S., et al. [21]	1	2021

Table S5. Level of evidence about Estrogen and Thyroid cancer.

Study	Level of evidence	Year
Sun, L.-M., et al. [22]	2	2020
Messuti, I., et al. [23]	3	2014
Kim, M., et al. [24]	2	2021
Rosenblatt, K. A., et al. [25]	3	2009

Table S6. Level of evidence about Autoimmune thyroid disease and Breast cancer.

Study	Level of evidence	Year
Ito, K et al. [26]	4	1975
Giani, C., et al. [27]	4	1996
Smyth, P. P., et al. [28]	4	1998
Turken, O., et al. [29]	4	2003
Giustarini, E., et al. [30]	4	2006
Jiskra, J., et al. [31]	4	2007
Graceffa, G., et al. [32]	3	2021
Kuijpens, J. L. P., et al. [17]	2	2005
Hedley, A. J., et al. [33]	3	1981
Pan, X.-F., et al. [34]	1	2020
Szychta, P., et al. [35]	4	2013

Table S7. Level of evidence about Oncogenic effects of the therapies for primary cancer.

Study	Level of evidence	Year
Ron, E., et al. [36]	3	1998
Metso, S., et al. [37]	2	2007
Ryödi, E., et al. [38]	2	2015
Kitahara, C. M., et al. [39]	3	2019
Melo, D. R., et al. [40]	3	2015
Silva-Vieira, M., et al. [41]	3	2017
Lin, C.-Y., et al. [42]	3	2016
Ko, K.-Y., et al. [43]	3	2015
Shim, S. R., et al. [44]	1	2021
de Groot, S., et al. [45]	4	2015
Kailajärvi, M., et al. [46]	4	2000

Kumar, N., et al. [47]	2	2004
Khan, M. A., et al. [48]	2	2015
Mortezaee, K., et al. [49]	1	2019
Rahu, K., et al. [50]	3	2013
Ivanov, V. K., et al. [51]	3	2002
Schaapveld, M., et al. [52]	3	2015
Grantzau, T., et al. [53]	2	2013
Akın, M., et al. [54]	3	2014
Choi, S. H., et al. [55]	2	2021
Darvish, L., et al. [56]	1	2018
Grantzau, Trine et al. [57]	1	2016
Reinertsen, K. V., et al. [58]	3	2009
Sechopoulos, I et al. [59]	4	2012
Yuan, M.-K., et al. [60]	4	2014

Table S8. Level of evidence about Genetic predisposition.

Study	Level of evidence	Year
Mucci, L. A., et al. [61]	2	2016
Zheng, G., et al. [62]	3	2017
Ngeow, J., et al. [63]	2	2014
Bennett, K. L., et al. [64]	4	2010

Table S9. Level of evidence about other factors and Breast cancer.

Study	Level of evidence	Year
Neuhouser, M. L., et al. [65]	2	2015
Ewertz, M., et al. [66]	3	2011
Engeland, A., et al. [67]	3	2006
Kitahara, C. M., et al. [68]	3	2012
He, Q., et al. [69]	4	2019
Hwang, Y., et al. [70]	4	2016
Rinaldi, S., et al. [71]	2	2012
Kim, H. J., et al. [72]	3	2013
Park, Y. M. M., et al. [73]	2	2021
Wang, T., et al. [74]	2	2021

2. Search criteria:

Search statement: (("Thyroid Neoplasms"[Mesh]) OR (((Thyroid Carcinoma*) OR (Thyroid Cancer*)) OR (Thyroid Neoplasm*))) AND (("Breast Neoplasms"[Mesh]) OR ((((((Breast Malignant Tumor*[Title/Abstract]) OR (Breast Cancer*[Title/Abstract])) OR (Breast Malignant Neoplasm*[Title/Abstract])) OR (Mammary Cancer*[Title/Abstract])) OR (Mammary Carcinoma*[Title/Abstract])) OR (Breast Carcinoma*[Title/Abstract])))

Database: Pubmed

Time limit: from the earliest literatue archived to Sept. 30th, 2022

Literature type: abstract, book, dataset, reference materials were excluded

After an electronic search of PubMed was conducted in September 2022 for literature published in English. 6749 literatures wrer found and the duplicates were removed. Then two researcheres screened the title and abstract and selected relevant literatures respectively. Finally, after reading full text of insterested literatures of each parts, we wrote manuscript and cited these literatures. The following terms were used, and relevant articles were considered as well: (thyroid cancer*), (breast cancer*), (genetic), (obesity), (second primary cancer).

3. Cowden Sydrome and Cowden Sydrome-like

OMIM graphical views of phenotype-gene relationships is in PDF file named OMIM_graphics.

3.1 Phenotypic Series:

Location	Phenotype	Inheritance	Phenotype mapping key	Phenotype MIM number	Gene/Locus	Gene/Locus MIM number
3q26.32	Cowden syndrome 5		3	615108	PIK3CA	171834
10q23.31	Cowden syndrome 4		3	615108	KLLN	612105
10q23.31	Lhermitte-Duclos disease	AD	3	615108	PTEN	601728
10q23.31	Cowden syndrome 1	AD	3	158350	PTEN	601728
14q32.33	Cowden syndrome 6		3	615109	AKT1	601728
14q32.33	?Cowden syndrome 7	AD	3	615109	SEC23B	610512

3.2 Clinical Synopsis:

INHERITANCE

- Autosomal dominant

GROWTH

Weight

- Obesity, increased risk of

HEAD & NECK

Head

- Progressive macrocephaly

Face

- 'Birdlike' facies (uncommon)
- Hypoplastic mandible (uncommon)
- Hypoplastic maxilla (uncommon)

Ears

- Hearing loss

Eyes
- Cataract
- Angioid streaks
- Myopia

Mouth
- Microstomia
- High-arched palate
- Scrotal tongue
- Oral papillomas

CARDIOVASCULAR
Vascular
- Vascular anomalies (50% of patients)
- Intracranial developmental venous anomalies

CHEST
Ribs Sternum Clavicles & Scapulae
- Pectus excavatum

Breasts
- Virginal hyperplasia
- Fibrocystic breast disease
- Gynecomastia in males
- Breast fibroadenomas

ABDOMEN
Gastrointestinal
- Hamartomatous polyps
- Colonic diverticula

GENITOURINARY
External Genitalia (Male)
- Hydrocele
- Varicocele

External Genitalia (Female)
- Vaginal cysts
- Vulvar cysts

Internal Genitalia (Female)

- Ovarian cysts
- Leiomyomas

SKELETAL

Spine

- Scoliosis
- Kyphosis

SKIN, NAILS, & HAIR

Skin

- Multiple facial papules
- Acral keratoses
- Palmoplantar keratoses
- Multiple skin tags
- Facial trichilemmomas
- Subcutaneous lipomas

NEUROLOGIC

Central Nervous System

- Seizure
- Intention tremor
- Lhermitte-Duclos disease
- Mental retardation, mild to moderate (in 12%)
- Psychomotor delay, mild to moderate
- Cerebellar gangliocytoma manifesting as seizure and tremor

ENDOCRINE FEATURES

- Goiter
- Thyroid adenoma
- Hyperthyroidism
- Hypothyroidism
- Thyroiditis
- Enhanced insulin sensitivity

IMMUNOLOGY

- Primary immunodeficiency (in some patients)
- Recurrent infections (in some patients)
- Opportunistic infections (in some patients)
- Hypogammaglobulinemia (in some patients)
- Lymphopenia (in some patients)
- Inverted CD4/CD8 T cell ratio (in some patients)
- Decreased memory B cells (in some patients)
- Decreased class-switched B cells (in some patients)

NEOPLASIA

- Breast cancer
- Ovarian carcinoma
- Cervical carcinoma
- Uterine adenocarcinoma
- Thyroid cancer (follicular cell)
- Transitional cell carcinoma of the bladder
- Meningioma
- Mucosal neuromas

MISCELLANEOUS

- Symptoms usually occur in adults
- Skin lesions are fully penetrant by second decade
- Skeletal abnormalities are variable
- Allelic to Bannayan-Riley-Ruvalcaba syndrome (153480), which has an earlier age at onset
- Approximately 80% of CS patients have PTEN mutations

MOLECULAR BASIS

- Caused by mutation in the phosphatase and tensin homolog gene (PTEN, 601728.0001)

1. Ron E, Curtis R, Hoffman DA, Flannery JT. Multiple primary breast and thyroid cancer. *Br J Cancer.* 1984;49(1):87-92.
2. Osterlind A, Olsen JH, Lynge E, Ewertz M. Second cancer following cutaneous melanoma and cancers of the brain, thyroid, connective tissue, bone, and eye in Denmark, 1943-80. *Natl Cancer Inst Monogr.* 1985;68:361-388.
3. Edhemović I, Volk N, Auersperg M. Second primary cancers following thyroid cancer in Slovenia. A population-based cohort study. *Eur J Cancer.* 1998;34(11):1813-1814.
4. Evans HS, Lewis CM, Robinson D, Bell CM, Møller H, Hodgson SV. Incidence of multiple primary cancers in a cohort of women diagnosed with breast cancer in southeast England. *Br J Cancer.* 2001;84(3):435-440.
5. Soerjomataram I, Louwman WJ, de Vries E, Lemmens VEPP, Klokman WJ, Coebergh JWW. Primary malignancy after primary female breast cancer in the South of the Netherlands, 1972-2001. *Breast Cancer Res Treat.* 2005;93(1):91-95.
6. Langballe R, Olsen JH, Andersson M, Mellemkjær L. Risk for second primary non-breast cancer in pre- and postmenopausal women with breast cancer not treated with chemotherapy, radiotherapy or endocrine therapy. *Eur J Cancer.* 2011;47(6):946-952.
7. Mellemkjaer L, Friis S, Olsen JH, et al. Risk of second cancer among women with breast cancer. *Int J Cancer.* 2006;118(9):2285-2292.
8. Kim C, Bi X, Pan D, et al. The risk of second cancers after diagnosis of primary thyroid cancer is elevated in thyroid microcarcinomas. *Thyroid.* 2013;23(5):575-582.

9. Nielsen SM, White MG, Hong S, et al. The Breast-Thyroid Cancer Link: A Systematic Review and Meta-analysis. *Cancer Epidemiol Biomarkers Prev.* 2016;25(2):231-238.
10. Tran T-V-T, Kitahara CM, de Vathaire F, Boutron-Ruault M-C, Journy N. Thyroid dysfunction and cancer incidence: a systematic review and meta-analysis. *Endocr Relat Cancer.* 2020;27(4):245-259.
11. Kapdi CC, Wolfe JN. Breast cancer. Relationship to thyroid supplements for hypothyroidism. *JAMA.* 1976;236(10):1124-1127.
12. Tosovic A, Becker C, Bondeson A-G, et al. Prospectively measured thyroid hormones and thyroid peroxidase antibodies in relation to breast cancer risk. *Int J Cancer.* 2012;131(9):2126-2133.
13. Hellevik AI, Asvold BO, Bjøro T, Romundstad PR, Nilsen TIL, Vatten LJ. Thyroid function and cancer risk: a prospective population study. *Cancer Epidemiol Biomarkers Prev.* 2009;18(2):570-574.
14. Journy NMY, Bernier M-O, Doody MM, Alexander BH, Linet MS, Kitahara CM. Hyperthyroidism, Hypothyroidism, and Cause-Specific Mortality in a Large Cohort of Women. *Thyroid.* 2017;27(8):1001-1010.
15. Kim EY, Chang Y, Lee KH, et al. Serum concentration of thyroid hormones in abnormal and euthyroid ranges and breast cancer risk: A cohort study. *Int J Cancer.* 2019;145(12):3257-3266.
16. Søgaard M, Farkas DK, Ehrenstein V, Jørgensen JOL, Dekkers OM, Sørensen HT. Hypothyroidism and hyperthyroidism and breast cancer risk: a nationwide cohort study. *Eur J Endocrinol.* 2016;174(4):409-414.
17. Kuijpens JLP, Nyklíctek I, Louwman MWJ, Weetman TAP, Pop VJM, Coebergh J-WW. Hypothyroidism might be related to breast cancer in post-menopausal women. *Thyroid.* 2005;15(11):1253-1259.
18. Ortega-Olvera C, Ulloa-Aguirre A, Ángeles-Llerenas A, et al. Thyroid hormones and breast cancer association according to menopausal status and body mass index. *Breast Cancer Res.* 2018;20(1):94.
19. Bach L, Kostev K, Schiffmann L, Kalder M. Association between thyroid gland diseases and breast cancer: a case-control study. *Breast Cancer Res Treat.* 2020;182(1):207-213.
20. Freitas PAVCJ, Vissoci GM, Pinto RM, Lajolo PP, Jorge PT. STUDY OF THE PREVALENCE OF AUTOIMMUNE THYROID DISEASE IN WOMEN WITH BREAST CANCER. *Endocr Pract.* 2016;22(1):16-21.
21. Chen S, Wu F, Hai R, et al. Thyroid disease is associated with an increased risk of breast cancer: a systematic review and meta-analysis. *Gland Surg.* 2021;10(1):336-346.
22. Sun L-M, Chung L-M, Lin C-L, Kao C-H. Uterine Fibroids Increase the Risk of Thyroid Cancer. *Int J Environ Res Public Health.* 2020;17(11).
23. Messuti I, Corvisieri S, Bardesono F, et al. Impact of pregnancy on prognosis of differentiated thyroid cancer: clinical and molecular features. *Eur J Endocrinol.* 2014;170(5):659-666.
24. Kim M, Kim BH, Lee H, et al. Thyroid cancer after hysterectomy and oophorectomy: a nationwide cohort study. *Eur J Endocrinol.* 2021;184(1):143-151.
25. Rosenblatt KA, Gao DL, Ray RM, et al. Oral contraceptives and the risk of all cancers combined and site-specific cancers in Shanghai. *Cancer Causes Control.* 2009;20(1):27-

34.
26. Ito K, Maruchi N. Breast cancer in patients with Hashimoto's thyroiditis. *Lancet.* 1975;2(7945):1119-1121.
27. Giani C, Fierabracci P, Bonacci R, et al. Relationship between breast cancer and thyroid disease: relevance of autoimmune thyroid disorders in breast malignancy. *J Clin Endocrinol Metab.* 1996;81(3):990-994.
28. Smyth PP, Shering SG, Kilbane MT, et al. Serum thyroid peroxidase autoantibodies, thyroid volume, and outcome in breast carcinoma. *J Clin Endocrinol Metab.* 1998;83(8):2711-2716.
29. Turken O, NarIn Y, DemIrbas S, et al. Breast cancer in association with thyroid disorders. *Breast Cancer Res.* 2003;5(5):R110-R113.
30. Giustarini E, Pinchera A, Fierabracci P, et al. Thyroid autoimmunity in patients with malignant and benign breast diseases before surgery. *Eur J Endocrinol.* 2006;154(5):645-649.
31. Jiskra J, Barkmanova J, Limanova Z, et al. Thyroid autoimmunity occurs more frequently in women with breast cancer compared to women with colorectal cancer and controls but it has no impact on relapse-free and overall survival. *Oncol Rep.* 2007;18(6):1603-1611.
32. Graceffa G, Scerrino G, Militello G, et al. Breast cancer in previously thyroidectomized patients: which thyroid disorders are a risk factor? *Future Sci OA.* 2021;7(5):FSO699.
33. Hedley AJ, Jones SJ, Spiegelhalter DJ, et al. Breast cancer in thyroid disease: fact or fallacy? *Lancet.* 1981;1(8212):131-133.
34. Pan X-F, Ma Y-J, Tang Y, Yu M-M, Wang H, Fan Y-R. Breast cancer populations may have an increased prevalence of thyroglobulin antibody and thyroid peroxidase antibody: a systematic review and meta-analysis. *Breast Cancer.* 2020;27(5):828-836.
35. Szychta P, Szychta W, Gesing A, Lewiński A, Karbownik-Lewińska M. TSH receptor antibodies have predictive value for breast cancer - retrospective analysis. *Thyroid Res.* 2013;6(1):8.
36. Ron E, Doody MM, Becker DV, et al. Cancer mortality following treatment for adult hyperthyroidism. Cooperative Thyrotoxicosis Therapy Follow-up Study Group. *JAMA.* 1998;280(4):347-355.
37. Metso S, Auvinen A, Huhtala H, Salmi J, Oksala H, Jaatinen P. Increased cancer incidence after radioiodine treatment for hyperthyroidism. *Cancer.* 2007;109(10):1972-1979.
38. Ryödi E, Metso S, Jaatinen P, et al. Cancer Incidence and Mortality in Patients Treated Either With RAI or Thyroidectomy for Hyperthyroidism. *J Clin Endocrinol Metab.* 2015;100(10):3710-3717.
39. Kitahara CM, Berrington de Gonzalez A, Bouville A, et al. Association of Radioactive Iodine Treatment With Cancer Mortality in Patients With Hyperthyroidism. *JAMA Intern Med.* 2019;179(8):1034-1042.
40. Melo DR, Brill AB, Zanzonico P, et al. Organ Dose Estimates for Hyperthyroid Patients Treated with (131)I: An Update of the Thyrotoxicosis Follow-Up Study. *Radiat Res.* 2015;184(6):595-610.
41. Silva-Vieira M, Carrilho Vaz S, Esteves S, et al. Second Primary Cancer in Patients with Differentiated Thyroid Cancer: Does Radioiodine Play a Role? *Thyroid.* 2017;27(8):1068-1076.
42. Lin C-Y, Lin C-L, Huang W-S, Kao C-H. Risk of Breast Cancer in Patients with Thyroid

Cancer Receiving or Not Receiving 131I Treatment: A Nationwide Population-Based Cohort Study. *J Nucl Med.* 2016;57(5):685-690.

43. Ko K-Y, Kao C-H, Lin C-L, Huang W-S, Yen R-F. (131)I treatment for thyroid cancer and the risk of developing salivary and lacrimal gland dysfunction and a second primary malignancy: a nationwide population-based cohort study. *Eur J Nucl Med Mol Imaging.* 2015;42(8):1172-1178.
44. Shim SR, Kitahara CM, Cha ES, Kim S-J, Bang YJ, Lee WJ. Cancer Risk After Radioactive Iodine Treatment for Hyperthyroidism: A Systematic Review and Meta-analysis. *JAMA Netw Open.* 2021;4(9):e2125072.
45. de Groot S, Janssen LGM, Charehbili A, et al. Thyroid function alters during neoadjuvant chemotherapy in breast cancer patients: results from the NEOZOTAC trial (BOOG 2010-01). *Breast Cancer Res Treat.* 2015;149(2):461-466.
46. Kailajärvi M, Ahokoski O, Virtanen A, Salminen E, Irjala K. Alterations in laboratory test results during adjuvant breast cancer treatment. *Clin Chem Lab Med.* 2000;38(5):443-451.
47. Kumar N, Allen KA, Riccardi D, et al. Fatigue, weight gain, lethargy and amenorrhea in breast cancer patients on chemotherapy: is subclinical hypothyroidism the culprit? *Breast Cancer Res Treat.* 2004;83(2):149-159.
48. Khan MA, Bhurani D, Agarwal NB. Alteration of Thyroid Function in Indian HER 2-Negative Breast Cancer Patients Undergoing Chemotherapy. *Asian Pac J Cancer Prev.* 2015;16(17):7701-7705.
49. Mortezaee K, Ahmadi A, Haghi-Aminjan H, et al. Thyroid function following breast cancer chemotherapy: A systematic review. *J Cell Biochem.* 2019;120(8):12101-12107.
50. Rahu K, Hakulinen T, Smailyte G, et al. Site-specific cancer risk in the Baltic cohort of Chernobyl cleanup workers, 1986-2007. *Eur J Cancer.* 2013;49(13):2926-2933.
51. Ivanov VK, Tsyb AF, Petrov AV, Maksioutov MA, Shilyaeva TP, Kochergina EV. Thyroid cancer incidence among liquidators of the Chernobyl accident. Absence of dependence of radiation risks on external radiation dose. *Radiat Environ Biophys.* 2002;41(3):195-198.
52. Schaapveld M, Aleman BMP, van Eggermond AM, et al. Second Cancer Risk Up to 40 Years after Treatment for Hodgkin's Lymphoma. *N Engl J Med.* 2015;373(26):2499-2511.
53. Grantzau T, Mellemkjær L, Overgaard J. Second primary cancers after adjuvant radiotherapy in early breast cancer patients: a national population based study under the Danish Breast Cancer Cooperative Group (DBCG). *Radiother Oncol.* 2013;106(1):42-49.
54. Akın M, Ergen A, Unal A, Bese N. Irradiation doses on thyroid gland during the postoperative irradiation for breast cancer. *J Cancer Res Ther.* 2014;10(4):942-944.
55. Choi SH, Chang JS, Byun HK, et al. Risk of Hypothyroidism in Women After Radiation Therapy for Breast Cancer. *Int J Radiat Oncol Biol Phys.* 2021;110(2):462-472.
56. Darvish L, Ghorbani M, Teshnizi SH, et al. Evaluation of thyroid gland as an organ at risk after breast cancer radiotherapy: a systematic review and meta-analysis. *Clin Transl Oncol.* 2018;20(11):1430-1438.
57. Grantzau T, Overgaard J. Risk of second non-breast cancer among patients treated with and without postoperative radiotherapy for primary breast cancer: A systematic review and meta-analysis of population-based studies including 522,739 patients. *Radiother Oncol.* 2016;121(3):402-413.
58. Reinertsen KV, Cvancarova M, Wist E, et al. Thyroid function in women after multimodal

treatment for breast cancer stage II/III: comparison with controls from a population sample. *Int J Radiat Oncol Biol Phys.* 2009;75(3):764-770.
59. Sechopoulos I, Hendrick RE. Mammography and the Risk of Thyroid Cancer. *American Journal of Roentgenology.* 2012;198(3):705-707.
60. Yuan M-K, Chang S-C, Hsu L-C, Pan P-J, Huang C-C, Leu H-B. Mammography and the Risk of Thyroid and Hematological Cancers: A Nationwide Population-based Study. *Breast Journal.* 2014;20(5):496-501.
61. Mucci LA, Hjelmborg JB, Harris JR, et al. Familial Risk and Heritability of Cancer Among Twins in Nordic Countries. *JAMA.* 2016;315(1):68-76.
62. Zheng G, Yu H, Hemminki A, Försti A, Sundquist K, Hemminki K. Familial associations of female breast cancer with other cancers. *Int J Cancer.* 2017;141(11):2253-2259.
63. Ngeow J, Stanuch K, Mester JL, Barnholtz-Sloan JS, Eng C. Second malignant neoplasms in patients with Cowden syndrome with underlying germline PTEN mutations. *J Clin Oncol.* 2014;32(17):1818-1824.
64. Bennett KL, Mester J, Eng C. Germline epigenetic regulation of KILLIN in Cowden and Cowden-like syndrome. *JAMA.* 2010;304(24):2724-2731.
65. Neuhouser ML, Aragaki AK, Prentice RL, et al. Overweight, Obesity, and Postmenopausal Invasive Breast Cancer Risk: A Secondary Analysis of the Women's Health Initiative Randomized Clinical Trials. *JAMA Oncol.* 2015;1(5):611-621.
66. Ewertz M, Jensen M-B, Gunnarsdóttir KÁ, et al. Effect of obesity on prognosis after early-stage breast cancer. *J Clin Oncol.* 2011;29(1):25-31.
67. Engeland A, Tretli S, Akslen LA, Bjørge T. Body size and thyroid cancer in two million Norwegian men and women. *Br J Cancer.* 2006;95(3):366-370.
68. Kitahara CM, Platz EA, Park Y, Hollenbeck AR, Schatzkin A, Berrington de González A. Body fat distribution, weight change during adulthood, and thyroid cancer risk in the NIH-AARP Diet and Health Study. *Int J Cancer.* 2012;130(6):1411-1419.
69. He Q, Sun H, Li F, Liang N. Obesity and risk of differentiated thyroid cancer: A large-scale case-control study. *Clin Endocrinol (Oxf).* 2019;91(6):869-878.
70. Hwang Y, Lee KE, Park YJ, et al. Annual Average Changes in Adult Obesity as a Risk Factor for Papillary Thyroid Cancer: A Large-Scale Case-Control Study. *Medicine.* 2016;95(9):e2893.
71. Rinaldi S, Lise M, Clavel-Chapelon F, et al. Body size and risk of differentiated thyroid carcinomas: findings from the EPIC study. *Int J Cancer.* 2012;131(6):E1004-E1014.
72. Kim HJ, Kim NK, Choi JH, et al. Associations between body mass index and clinico-pathological characteristics of papillary thyroid cancer. *Clin Endocrinol (Oxf).* 2013;78(1):134-140.
73. Park YMM, Bookwalter DB, O'Brien KM, Jackson CL, Weinberg CR, Sandler DP. A prospective study of type 2 diabetes, metformin use, and risk of breast cancer. *Ann Oncol.* 2021;32(3):351-359.
74. Wang T, Farvid MS, Kang JH, et al. Diabetes Risk Reduction Diet and Survival after Breast Cancer Diagnosis. *Cancer Res.* 2021;81(15):4155-4162.
